# Supplementary material for: Toward integrating clinical and non-clinical associates of suicidality to inform potential intervention points among youth in Nairobi metropolitan, Kenya
Source: Glob Ment Health (Camb). 2026 Feb 6;13:e33. doi: 10.1017/gmh.2026.10148 (PMC12951344; doi:10.1017/gmh.2026.10148)
Supplement: Ndetei et al. supplementary material [file S2054425126101484sup001.docx]

**Supplementary file 1**

## COLUMBIA- SUICIDE SEVERITY RATING SCALE (C-SSRS)

| ***SECTION A: SUICIDAL IDEATION*** | | |
| --- | --- | --- |
| *If both question 1 and 2 are negative, proceed to “Suicidal Behavior” section. If the answer to* *question 2 is “yes”, go to questions 3, 4 and 5. If the answer to question 1 and/or 2 is “yes”, complete* *“Intensity of Ideation” section below.* | **Lifetime** | **Past 1**  **month** |
| **1. Wish to be Dead**    ***Have you wished you were dead or wished you could go to sleep and not wake up?***    If yes, describe: | **Yes No**  **□ □** | **Yes No**  **□ □** |
| **2. Non-Specific Active Suicidal Thoughts**    ***Have you actually had any thoughts of killing yourself?***    If yes, describe: | **Yes No**  **□ □** | **Yes No**  **□ □** |
| **3. Active Suicidal Ideation with Any Methods (Not Plan) without Intent to Act**    ***Have you been thinking about how you might do this?***    If yes, describe: | **Yes No**  **□ □** | **Yes No**  **□ □** |
| **4. Active Suicidal Ideation with Some Intent to Act, without Specific Plan**    ***Have you had these thoughts and had some intention of acting on them?***    If yes, describe: | **Yes No**  **□ □** | **Yes No**  **□ □** |
| **5. Active Suicidal Ideation with Specific Plan and Intent**    ***Have you started to work out or worked out the details of how to kill yourself? Do you intend to carry out this plan?***    If yes, describe: | **Yes No**  **□ □** | **Yes No**  **□ □** |
| ***SECTION B: INTENSITY OF IDEATION*** | | |
| *The following features should be rated with respect to the most severe type of ideation (i.e., 1-5 from above, with 1 being* *the least severe and 5 being the most severe). Ask about time he/she was feeling the most suicidal.*    Lifetime - ***Most Severe Ideation:***  ***Type # (1-5) Description of Ideation***    Recent ***- Most Severe Ideation:***  ***Type # (1-5) Description of Ideation*** | Most  Severe | Most  Severe |
|  |  |  |
|  |  |  |
| **Frequency**  ***How many times have you had these thoughts?***  (1) Less than once a week (2) Once a week (3) 2-5 times in week (4) Daily or almost daily (5) Many times each day |  |  |
| **Duration**  ***When you have the thoughts how long do they last?***   1. Fleeting - few seconds or minutes (4) 4-8 hours/most of day 2. Less than 1 hour/some of the time (5) More than 8 hours/persistent or continuous 3. 1-4 hours/a lot of time |  |  |
| **Controllability**  ***Could/can you stop thinking about killing yourself or wanting to die if you want to?***   1. Easily able to control thoughts (4) Can control thoughts with a lot of difficulty 2. Can control thoughts with little difficulty (5) Unable to control thoughts 3. Can control thoughts with some difficulty (0) Does not attempt to control thoughts |  |  |
| **Deterrents**  ***Are there things - anyone or anything (e.g., family, religion, pain of death) - that stopped you from wanting to die or acting on thoughts of committing suicide?***   1. Deterrents definitely stopped you from attempting suicide (4) Deterrents most likely did not stop you 2. Deterrents probably stopped you (5) Deterrents definitely did not stop you (3) Uncertain that deterrents stopped you (0) Does not apply |  |  |
| **Reasons for Ideation**  ***What sort of reasons did you have for thinking about wanting to die or killing yourself? Was it to end the pain or stop the way you were feeling (in other words you couldn’t go on living with this pain or how you were feeling) or was it to get attention, revenge or a reaction from others? Or both?***   1. Completely to get attention, revenge or a reaction from others (4) Mostly to end or stop the pain (you couldn’t go on 2. Mostly to get attention, revenge or a reaction from others living with the pain or how you were feeling) 3. Equally to get attention, revenge or a reaction from others (5) Completely to end or stop the pain (you couldn’t go on and to end/stop the pain living with the pain or how you were feeling) (0) Does not apply |  |  |

| ***SECTION C: SUICIDAL BEHAVIOR*** | **Lifetime** | **Past 3 months** |
| --- | --- | --- |
| **Actual Attempt:**     1. ***Have you*** 🌕 ***made a suicide attempt?*** 2. ***Have you done anything to harm yourself?*** 3. ***Have you done anything dangerous where you could have died?***  ***What did you do?*** 4. ***Did you do this as a way to end your life?*** 5. ***Did you want to die (even a little) when you did this?*** 6. ***Were you trying to end your life when you did this?*** 7. ***Did you think it was possible you could have died from this?*** 8. ***Did you do it purely for other reasons / without ANY intention of killing yourself (like to relieve stress, feel better, get sympathy, or get something else to happen)?*** *(*Self-Injurious Behavior without   suicidal intent)    If **yes**, describe: ……………………………………………………………………………………………………………………… …………………………………………………………………………………………………………………………………..        **Have you engaged in Non-Suicidal Self-Injurious Behavior?** | Yes No  1□ □  2□ □  3□ □  4□ □  5□ □  6□ □  7□ □  8□ □      Total # of  Attempts      **Yes No**  **□ □** | Yes No  1□ □  2□ □  3□ □  4□ □  5□ □  6□ □  7□ □  8□ □        Total # of  Attempts      **Yes No**  **□ □** |
| **Interrupted Attempt:**    ***Has there been a time when you started to do something to end your life but someone or something stopped you before you actually did anything?***    If yes, describe: | **Yes No**  **□ □**          Total # of interrupted | **Yes No**  **□ □**          Total # of interrupted |
| **Aborted or Self-Interrupted Attempt:**    ***Has there been a time when you started to do something to try to end your life but you stopped yourself before you actually did anything?***    If yes, describe: | **Yes No**  **□ □**    Total # of aborted or self- interrupted | **Yes No**  **□ □**    Total # of aborted or  self-  interrupted |
| **Preparatory Acts or Behavior:**    ***Have you taken any steps towards making a suicide attempt or preparing to kill yourself?***    If yes, describe: | **Yes No**  **□ □**    Total # of preparatory acts | **Yes No**  **□ □**    Total # of preparatory acts |

| **Instructions:** *Check all that apply to you* | |  | |
| --- | --- | --- | --- |
|  | | Tick here  (🗸 ) |  |
| **SECTION D: Activating Events (Recent**) | |  |  |
|  | Recent loss or other significant negative event |  |  |
|  | Describe: |  |  |
|  | Current or pending isolation or feeling alone |  |  |
| **SECTION E: Treatment History** | |  |  |
|  | Previous psychiatric diagnoses and treatments |  |  |
|  | Hopeless or dissatisfied with treatment |  |  |
|  | Noncompliant with treatment |  |  |
|  | Not receiving treatment |  |  |
| **SECTION F: Protective Factors (Recent**) | |  |  |
|  | Identifies reasons for living |  |  |
|  | Responsibility to family or others; living with family |  |  |
|  | Supportive social network or family |  |  |
|  | Fear of death or dying due to pain and suffering |  |  |
|  | Belief that suicide is immoral, high spirituality |  |  |
|  | Engaged in work or school |  |  |
|  | Other Protective Factors |  |  |
